# Analgesics and ASH medications in workers increase the risk of disability pension and mortality: prospective cohort

**DOI:** 10.1093/eurpub/ckad064

**Published:** 2023-06-06

**Authors:** Lars Louis Andersen, Jonas Vinstrup, Joaquín Calatayud, Rubén López-Bueno, Thomas Clausen, Claus Manniche

**Affiliations:** National Research Centre for the Working Environment, Copenhagen, Denmark; Department of Health Science and Technology, Aalborg University, Aalborg, Denmark; National Research Centre for the Working Environment, Copenhagen, Denmark; National Research Centre for the Working Environment, Copenhagen, Denmark; Exercise Intervention for Health Research Group (EXINH-RG), Department of Physiotherapy, University of Valencia, Valencia, Spain; National Research Centre for the Working Environment, Copenhagen, Denmark; Department of Physical Medicine and Nursing, University of Zaragoza, C. de Pedro Cerbuna, Zaragoza, Spain; National Research Centre for the Working Environment, Copenhagen, Denmark; Department of Occupational and Environmental Medicine, Institute of Clinical Research, Odense University Hospital, University of Southern Denmark, Odense, Denmark

## Abstract

**Background:**

Relying on medication for musculoskeletal and mental disorders are common, but may have long-term consequences. This study investigates whether use of analgesics and anxiolytic/sedative/hypnotic (ASH) medication increases the risk of disability pension and mortality.

**Methods:**

After completing a survey in 2005, 7773 female eldercare workers were followed for 11 years in a national register. We estimated hazard ratios (HRs) for disability pension and mortality from using analgesics and ASH.

**Results:**

During follow-up, 10.3% obtained disability pension and 2.4% died. For use of analgesics, a frequency-response association for the risk of disability pension existed with HR’s (95% confidence interval) of 1.30 (1.07–1.57), 2.00 (1.62–2.46) and 3.47 (2.69–4.47) for monthly, weekly and daily use, respectively. For ASH, an increased risk of disability pension also existed (HR’s between 1.51 and 1.64). For mortality risk, only daily use of analgesics and ASH remained significant. Population attributable fractions of analgesics and ASH, respectively, were 30% and 3% for disability pension and 5% and 3% for mortality.

**Conclusions:**

Frequent use of analgesics and ASH medication in workers increase the risk of disability pension and early death. Better management of musculoskeletal and mental health conditions, without excessive medication use, is necessary.

## Additional content

An author video to accompany this article is available at: https://oup.cloud.panopto.eu/Panopto/Pages/Viewer.aspx?id=27aaa92a-8588-4fde-9887-affc01219cd1&start=0

## Introduction

The Global Burden of Disease Study shows that common musculoskeletal- and mental disorders are leading causes of years lived with disability.^1^ Country-specific data from Denmark show that low-back pain, neck pain, arthritis and depression contribute markedly to the total burden of disease, in terms of work absence, lost work productivity and healthcare expenses.^2^ In adult workers, these conditions increase the risk of sickness absence and early involuntary exit from the labour market.^3–5^ Because work is a fundamental part of our lives, economically as well as socially, the majority of people attempts to continue working in spite of such health problems. In many cases, relying on medicine to remain active at the labour market despite musculoskeletal- or mental issues, poses a tempting—and sometimes necessary—solution. While medicine developed during the last century undoubtedly have saved and prolonged millions of lives, the World Health Organization estimates that more than half of all medicine is prescribed, dispensed or sold inappropriately.^6^

Although medication for musculoskeletal pain provides efficient short-term pain relief, frequent use often results in diminishing effects, addictive behaviour, and worsening of symptoms.^7^^,^^8^ Furthermore, pain medication does not improve functional outcomes or accelerate return to work in injured workers.^7^ In this category of pain medication, opioids are a class of drugs demanding special attention. Worldwide, in 2017, more than 40 million people were addicted to opioid analgesics, and more than 100 000 died from opioid overdose.^9^ The Centers for Disease Control and Prevention in the USA reported 34.1 per 100 000 persons overdose deaths due to drugs in 2021, of which 28.1 per 100 000 persons were due to opioids.^10^ Additionally, research from Canada and the USA show increased mortality from prescription as well as non-prescription opioids^11^^,^^12^; highlighting why ‘The Burden of Opioid-Related Mortality’ is a recognized term. Likewise, in Europe, the use of opioids has markedly increased during recent years,^13^^,^^14^ indicating a growing, worldwide trend of excessive use of pain medication. Lastly, a recent systematic review with meta-analysis reported increased risk of premature mortality from excessive use of opioids,^15^ highlighting the urgent need for improved management and post-treatment follow-up.

Along similar lines, the use of prescription medication for common mental disorders also warrants further attention. Not unlike pain medication, treatment with anxiolytic/sedative/hypnotic (ASH) medication is highly efficacious when properly prescribed and utilized, while excessive use shows diminishing effect over time and often result in addictive behaviour and several adverse effects.^16–19^ A systematic review with meta-analysis, including 25 studies with a total of more than 2 million patients, showed increased mortality risk from ASH medication.^20^ Altogether, the potential benefits of medication for common musculoskeletal- and mental disorders are questionable, and may—in several cases—be overwhelmingly outweighed by the adverse effects associated with chronic excessive use.

While some of the stronger medications are prescription-based, many over-the-counter medications (e.g. paracetamol and non-steroidal anti-inflammatory drugs) are also widely used,^21^ which complicates the interpretation of research studies based solely on registers. Thus, a combination of surveys and registers is vital to capture the full scope of these challenges. In Denmark, disability benefits are granted and registered in case of full or partial loss of work ability.^22^ For the purpose of research studies, data on disability pension from national registers thereby poses a valuable objective measure of deteriorated work ability.

Therefore, this study investigated the association of using analgesics and ASH medication with the risk of disability pension and mortality among healthcare workers. We hypothesized that use of these medications would be prospectively associated with increased risk of disability pension and mortality.

## Methods

### Population

In this prospective cohort study with 11-year register follow-up, baseline data collection took place in 2005. A total of 12 744 healthcare workers in eldercare received the baseline questionnaire, of which 9949 (78%) responded. To obtain a homogenous sample for the present analyses, we excluded male respondents (*n* = 234), workers who were not directly engaged in care services (*n* = 1,021), and workers with previous long-term sickness absence (*n* = 594), resulting in 8137 healthcare workers. Finally, we excluded those not responding to the specific questions about medication, resulting in a final sample size of 7773. This comprised social and healthcare assistants, social and healthcare helpers, other care staff with no or short-term education and registered nurses/therapists. Table 1 shows the baseline characteristics of the included sample of female healthcare workers without previous long-term sickness absence. The reporting of this study follows the STROBE guidelines for observational cohort studies.

**Table 1:** Descriptive baseline characteristics of the 7773 female healthcare workers without previous long-term sickness absence

	*N*	Mean	SD	%
**Age**	7773	45.1	10.0	
**Smoking**				
**Yes**	2815			36.2
**No**	4958			63.8
**Physical activity level during leisure**				
**Low**	3578			46.0
**Moderate**	3839			49.4
**High**	356			4.6
**Body mass index (kg/m^2^)**	7773	24.9	4.3	
**Physical exertion during work (1–7)**	7773	3.8	1.2	
**Psychosocial work factors (0–100)**				
**Emotional demands**	7773	45.8	18.4	
**Influence at work**	7773	45.2	20.5	
**Role conflicts**	7773	41.4	15.6	
**Quality of leadership**	7773	57.2	21.5	
**Number of body regions musculoskeletal pain >30 days**				
**0**	4648			59.8
**1**	1921			24.7
**2**	896			11.5
**3**	308			4.0
**Depressive symptoms (major depressive inventory, 0–46)**	7773	6.9	5.8	

### Predictors

Three questions assessed the frequency of use of medication: ‘Have you within the last 3 months used (1) pain medication (including headache pills), (2) sedatives, anxiolytics and (3) sleep medication’. In the present analyses, questions 2 and 3 were merged (highest frequency of the two), as these types of medications are often similar in effect and use. The response options for each were ‘(1) daily, (2) one or more times a week, (3) one or more times a month and (4) never or seldom’.

### Outcome

We used the Danish Register for Evaluation of Marginalization (DREAM) to obtain reliable information about registered disability benefit payments and mortality.^23^ This register contains week-to-week information about employment, education, sickness absence, granted disability benefits, mortality etc. for Danish residents. The outcome measures of the present study were therefore free from loss to follow-up. The municipality decides whether a person is entitled to disability benefits and only grants this in case of permanent full or partial loss of workability. As a starting point, the person must participate in a work ability (‘work-readiness’) enhancement program. In the assessment, the municipality considers various factors such as education, work experience, and health. To assess workability of the person, the municipality creates a rehabilitation plan in collaboration with the person. The plan describes the citizen’s resources and opportunities in relation to the demands of the labour market. Disability benefits include (i) full disability pension, i.e. complete dropout of the labour market, or (ii) disability benefits with partial work or work on certain conditions. Thus, this includes full disability pension, sheltered jobs, flex jobs and variants hereof (13 categories of disability benefits). As all of these categories document loss of workability, we defined ‘disability pension’ as receiving any type of registered disability benefit. In DREAM, mortality is registered as a single category without cause of death.

### Confounders

Potential confounders included age (continuous variable) and education (highest completed occupational education, e.g. social and healthcare assistant, social and healthcare helper, nurse, nurse aide, therapist, none). Work environmental factors included perceived physical exertion during work,^24^ and four psychosocial dimensions from the Copenhagen Psychosocial Questionnaire (i.e. emotional demands, influence at work, role conflicts and quality of leadership).^25^^,^^26^ Lifestyle factors included body mass index (BMI) (kg/m^2^, continuous variable), smoking status (dichotomous variable depicting smoker/non-smoker), and leisure-time physical activity (low, moderate and high level).^27^ Health-related factors included number of body regions with musculoskeletal pain in the low back, neck/shoulders and/or knees for more than 30 days during the last year,^28^ and depressive symptoms (Major Depressive Inventory, scoring 0–50).^29^ These confounders were chosen, as they have previously been associated with sickness absence or poor health.

### Statistical analyses

Using the Cox proportional hazards model (PHREG procedure of SAS 9.4, SAS Institute, Cary, NC, USA) we estimated hazard ratios (HRs) and 95% confidence intervals (95% CIs) for receiving disability pension and dying, respectively. This is a survival analysis used to model the time to an event. Follow-up in the DREAM register was until week 26 of 2016 (i.e. 11 years) or until censoring, which—in the case of disability pension as outcome variable—occurred for voluntary early retirement pension, state pension or emigration. Furthermore, for disability pension as outcome, death was included as competing risk according to the cumulative incidence function of Fine and Gray.^30^ In the case of death as outcome variable, censoring occurred for emigration, with no competing risks included. When an individual had a registered disability benefit payment or died in any given week within the follow-up period, the survival times were non-censored and referred to as event times. Multiple imputation replaced missing covariates (i.e. those described in the Confounders section). The predictor variables were use of analgesics and ASH (mutually adjusted). Model 1 (minimally adjusted) was controlled for age, education and type of medication and Model 2 (fully adjusted) was controlled for age, education, type of medication, work factors, lifestyle factors and health-related factors. On an exploratory basis, we also tested possible interactions with age.

Finally, population attributable fractions (PAFs) were calculated for the two types of medication, which expresses the contribution of a risk factor to the outcome. The PAF calculation was based on the HRs and proportions exposed (Pe) from the fully adjusted model. PAF (%) was calculated as ∑ Pe (HRe − 1)/(∑ Pe (HRe − 1) + 1) * 100%. To avoid overestimation, only the estimates of significant HR’s were used, i.e. non-significant HR’s were set to the value of ‘1’ in the calculation.

## Results

Table 1 shows baseline characteristics of the 7773 female healthcare workers without previous long-term sickness absence. The mean age of the study population was 45.1 years. For lifestyle factors, mean BMI was in the normal range, about one-third were smokers and the majority had moderate or low levels of leisure time physical activity. For health-related factors, about 40% had experienced pain for more than 30 days during the previous year in one or more body regions, and the mean Major Depressive Inventory score was 6.9.

During 11 years follow-up, 10.3% (*n* = 801) was granted a disability pension and 2.4% (*n* = 187) died. Analgesics and ASH, respectively, were used daily by 6.4% and 2.4% and weekly by 17.5% and 1.0% of the workers.

Table 2 shows the risk of disability pension from use of analgesics and ASH. For use of analgesics, a frequency–response association was found with HR’s (95% CI) of 1.30 (1.07–1.57), 2.00 (1.62–2.46), and 3.47 (2.69–4.47) for monthly, weekly and daily use, respectively. Likewise, for ASH, a significantly increased risk of disability pension was observed, although not in a frequency-response manner (HR’s between 1.51 and 1.64). PAF’s for disability pension from use of analgesics and ASH, respectively, were 30% and 3%. Use of medicine and age did not interact in the risk of disability pension (analgesics by age, *P* = 0.24; ASH by age, *P* = 0.55).

**Table 2:** During 11-year follow-up 804 (10.3%) of the 7773 healthcare workers were granted a disability pension

			Events		Hazard ratio (95% CI)		
	*n*	%	*n*	%	Model 1	Model 2	PAF^a^
**Analgesics**							
** 1. Never/seldomly**	3145	40.5	207	6.6	1	1	
** 2. Monthly**	2773	35.7	242	8.7	1.39 (1.15–1.67)	1.30 (1.07–1.57)	30%
** 3. Weekly**	1361	17.5	207	15.2	2.36 (1.93–2.89)	2.00 (1.62–2.46)	
** 4. Daily**	494	6.4	148	30.0	4.49 (3.57–5.63)	3.47 (2.69–4.47)	
**ASH**							
** 1. Never/seldomly**	7338	94.4	698	9.5	1	1	
** 2. Monthly**	175	2.3	39	22.3	1.85 (1.31–2.61)	1.60 (1.14–2.25)	3%
** 3. Weekly**	77	1.0	21	27.3	1.91 (1.22–2.99)	1.64 (1.04–2.58)	
** 4. Daily**	183	2.4	46	25.1	1.87 (1.34–2.62)	1.51 (1.06–2.14)	

*Note*: The table shows hazard ratios and population attributable fraction (PAF) for disability pension from use of analgesics and anxiolytic/sedative/hypnotic (ASH) medication. The first two columns show the number and column percentage of workers never using medicine to using medicine on a daily basis. The third and fourth columns show the number and row percentage of disability pension events in each of these groups.

Model 1: Adjusted for age, education and type of medication.

Model 2: Model 1 + psychosocial and physical work environment, education, lifestyle (physical activity, smoking and BMI), musculoskeletal pain and depressive symptoms.

a Population Attributable Fraction (PAF) estimated for significant (*P* < 0.05) HR’s of Model 2.

Table 3 shows the mortality risk from use of analgesics and ASH. For both types of medication, only daily use remained significant, with HR’s of 1.85 (1.10–3.12) for analgesics and 2.44 (1.37–4.35) for ASH. A sensitivity analysis excluding those who died during the first 104 weeks (2 years) after baseline did not change the results; HR’s of 1.77 (1.03–3.04) for analgesics and 2.67 (1.49–4.77) for ASH (not shown in Table 3). PAF’s for mortality from use of analgesics and ASH, respectively, were 5% and 3%. Analgesics and age did not interact in the risk of mortality (*P* = 0.72). However, ASH and age interacted in the risk of mortality (*P* = 0.02). In workers <50 years, age-stratified analyses showed HR’s of 4.44 (1.64–12.00), 1.78 (0.23–13.90) and 3.75 (1.37–10.27) for monthly, weekly and daily use of ASH, respectively, and for workers 50 years or older, HR’s of 1.02 (0.41–2.55), 1.62 (0.49–5.35) and 2.06 (1.02–4.19) were found.

**Table 3:** During 11-year follow-up 189 (2.4%) of the 7773 healthcare workers died

			Events		Hazard ratio (95% CI)		
	*n*	%	*n*	%	Model 1	Model 2	PAF^a^
**Analgesics**							
** 1. Never/seldomly**	3145	40.5	70	2.2	1	1	
** 2. Monthly**	2773	35.7	54	2.0	1.09 (0.76–1.56)	1.10 (0.76–1.59)	5%
** 3. Weekly**	1361	17.5	38	2.8	1.38 (0.92–2.07)	1.45 (0.94–2.22)	
** 4. Daily**	494	6.4	27	5.5	1.79 (1.12–2.88)	1.85 (1.10–3.12)	
**ASH**							
** 1. Never/seldomly**	7338	94.4	160	2.2	1	1	
** 2. Monthly**	175	2.3	10	5.7	1.94 (1.01–3.71)	1.71 (0.88–3.30)	3%
** 3. Weekly**	77	1.0	4	5.2	1.56 (0.57–4.29)	1.51 (0.54–4.22)	
** 4. Daily**	183	2.4	15	8.2	2.50 (1.42–4.38)	2.44 (1.37–4.35)	

*Note*: The table shows hazard ratios and population attributable fraction (PAF) for mortality from use of analgesics and anxiolytic/sedative/hypnotic (ASH) medication. The first two columns show the number and column percentage of workers never using medicine to using medicine on a daily basis. The third and fourth columns show the number and row percentage of mortality events in each of these groups.

Model 1: Adjusted for age, education and type of medication.

Model 2: Model 1 + psychosocial and physical work environment, education, lifestyle (physical activity, smoking and BMI), musculoskeletal pain and depressive symptoms.

a Population Attributable Fraction (PAF) estimated for significant (*P* < 0.05) HR’s of Model 2 (non-sign HR’s set to 1).

## Discussion

Our study found that use of analgesics and ASH among female healthcare workers is prospectively associated with increased risk of disability pension and mortality during an 11-year follow-up period. Overall, our findings on the outcome of mortality agree with prior research performed in Western countries and in different occupational groups. Additionally, our results add important insight to a scarce body of research on the prospective association of analgesics and ASH with disability pension.

Before discussing possible implications of the present findings, we will address a number of limitations and strengths of the study. Using self-reported use of medication as predictor serves as both a strength and limitation. Many types of analgesics are available over the counter and information about individual use is therefore not included in Danish registers, nor in registers of any other countries. A primary strength of the study is that self-reported (survey) use of medication captures total use, i.e. the combination of over-the-counter and prescription medication. A limitation is that we were not able to discriminate between different subgroups of medication within analgesics and ASH. Furthermore, as we only have information about use of medicine at baseline, and not during follow-up, we are unable to quantify whether use of analgesics and ASH medication changed over time and how this may have influenced the outcomes. Generally, self-reports are prone to recall bias in terms of frequency of use and type of medication. However, as both outcome measures—disability pension and mortality—came from a high-quality national register, we are certain of the robustness of the presented associations. Likewise, to avoid that severe or life-threatening disease influenced the main results, we excluded those with long-term sickness absence prior to baseline. Additionally, a sensitivity analysis excluding those who died during the first 2 years of follow-up did not change the results. We also controlled for a multitude of possible confounders, including those related to work, lifestyle and health. Furthermore, we included a rather homogenous sample of female healthcare workers, which reduces the risk of residual confounding from e.g. educational- and socioeconomic factors. Although causal inferences can never be drawn from observational studies, the present study underscores the strong prognostic value of self-reported use of analgesics and ASH medication for long-term adverse health outcomes. Furthermore, the present findings resonate with the previous studies described in the Introduction, investigating the hazardous influence of analgesics or ASH medication for long-term health outcomes.

There are several implications of the present findings. First, the strong prognostic value of self-reported use of medication suggests that this can add valuable information to research studies, compared with solely obtaining information about prescription-based medication from registers. Future research studies should therefore consider prioritizing detailed use of type, amount and number of different medications over a prolonged period, to allow for results applicable to real-world scenarios. Second, healthcare providers should be attentive about possible medication misuse, even lighter cases, as some patients may enter a negative spiral and end up with addictive behaviour.^7^ The decision to prescribe or not prescribe analgesics and ASH medication may be challenging for medical doctors, particularly when balancing the competing concerns of providing effective pain management while minimizing the potential risks of addiction, overdose, and other adverse events. This highlights the importance of evidence-based guidelines to guide the decision-making process. Ultimately, the decision to prescribe analgesics and ASH medication should be based on a careful assessment of the patient’s needs, medical history, and risk factors, taking into account both the benefits and potential harms of these medications. Third, workers and workplaces should be aware of possible negative health consequences of inappropriate use of medication and be provided with alternatives, e.g. workplace physical exercise for pain relief.^31^ For example, micro-exercise—consisting of strengthening exercises performed at the workplace for 10 min three times a week—can reduce musculoskeletal pain and prevent sickness absence.^32^ The latter may be achieved through general public education or specified workplace campaigns, focusing on healthy ways to manage musculoskeletal- and mental disorders without excessive use of medication. Ensuring a good ergonomic and psychosocial work environment is also important to prevent musculoskeletal and mental disorders.^26^^,^^33^^,^^34^ An alternative to cope could for example be job redesign interventions to ease the physical and mental work demands.

## Conclusions

Frequent use of analgesics and ASH medication in workers is prospectively associated with premature exit from the labour market and early death, hereby serving as important early warning signs. A large, unexplored potential exists for better prevention and management of musculoskeletal- and mental health conditions, without excessive use of medication.

## Ethical approval

According to Danish law, questionnaire and register-based studies do not need approval by an ethical committee nor informed consent (https://nationaltcenterforetik.dk/ansoegerguide/overblik/hvad-skal-jeg-anmelde).

## Data Availability

The authors encourage collaboration and use of the data by other researchers. Due to the Danish data protection legislation, micro data cannot be made publicly available. Researchers interested in using the data for scientific purposes should contact Prof. Lars L. Andersen, lla@nfa.dk.
